# Detection of the Phenicol–Oxazolidinone Resistance Gene *poxtA* in *Enterococcus faecium* and *Enterococcus faecalis* from Food-Producing Animals during 2008–2018 in Korea

**DOI:** 10.3390/microorganisms8111839

**Published:** 2020-11-23

**Authors:** Seok-Hyeon Na, Dong-Chan Moon, Mi-Hyun Kim, Hee-Young Kang, Su-Jeong Kim, Ji-Hyun Choi, Abraham-Fikru Mechesso, Soon-Seek Yoon, Suk-Kyung Lim

**Affiliations:** Bacterial Disease Division, Animal and Plant Quarantine Agency, 177 Hyeksin 8-ro, Gimcheon-si, Gyeongsangbuk-do 39660, Korea; nash8090@korea.kr (S.-H.N.); ansehdcks@korea.kr (D.-C.M.); kimmh940301@naver.com (M.-H.K.); kanghy7734@korea.kr (H.-Y.K.); kimsujeong27@gmail.com (S.-J.K.); wlgus01@korea.kr (J.-H.C.); abrahamf@korea.kr (A.-F.M.); yoonss24@korea.kr (S.-S.Y.)

**Keywords:** clonal complex 17, *Enterococcus*, linezolid resistance, *poxtA*

## Abstract

We aimed to investigate the presence of the phenicol–oxazolidinone resistance gene *poxtA* in linezolid-resistant enterococci from food-producing animals and analyze its molecular characteristics. We collected 3941 *Enterococcus faecium* and 5088 *E. faecalis* isolates from all provinces of South Korea from 2008 to 2018. We found linezolid resistance in 0.79% (94/3941) of *E. faecium* and 1.22% (62/5088) of *E. faecalis* isolates. Overall, 23.1% (36/156) of the linezolid-resistant isolates had the *poxtA* gene, including 31 *E. faecium* and five *E. faecalis* isolates. The *poxtA*-positive enterococci were mainly isolated from chicken (86.1%; 26/36). Fifteen *poxtA*-harboring isolates co-carried another linezolid-resistance gene, *optrA*. Eight *E. faecium* isolates had an N130K mutation in the ribosomal protein L4, while no mutations were observed in *E. faecalis* isolates. The *poxtA* gene was transferred into 10 enterococci by conjugation. Multi-locus sequence typing (MLST) and pulsed-field gel electrophoresis (PFGE) analysis indicated that *poxtA*-carrying isolates were heterogeneous. Three *E. faecium* isolates belonged to CC17 (ST32, ST121, and ST491). To our knowledge, this is the first report on the *poxtA* gene in Korea. Prudent use of antimicrobials and active surveillance on antimicrobial resistance are urgently needed to reduce the risk of dissemination of the linezolid-resistant isolates in humans and animals.

## 1. Introduction

Oxazolidinones, including linezolid and tedizolid, have been used for the treatment of severe bacterial infections caused by clinically important Gram-positive pathogens, such as vancomycin-resistant enterococci (VRE) and methicillin-resistant *Staphylococcus aureus* (MRSA) [1]. The use of linezolid in veterinary medicine is not globally approved. However, the emergence of linezolid resistance from non-human origin has been reported in several countries, including Korea [1,2,3].

Linezolid is known to interfere with the peptidyltransferase site of the bacterial ribosome. This leads to a disruption of protein synthesis and inhibition of bacterial growth [4]. Linezolid resistance has been associated with mutations in the domain V of 23S rRNA; mutations in the ribosomal proteins L3, L4, and L22; and the acquisition of resistance genes such as the multi-resistance gene *cfr* and ABC-F type ribosomal protection protein optrA [1,5]. The most recently described ABC-F transporter gene, *poxtA*, confers reduced susceptibility to phenicols, oxazolidinones, and tetracyclines. However, the *poxtA* gene cloned into three heterologous hosts (*Escherichia coli*, *Staphylococcus aureus*, and *Enterococcus faecalis*) induced up to a 4-fold increase in the minimum inhibitory concentration (MIC) of tetracyclines with concentrations much lower than the MIC breakpoints [6]. Thus, the *poxtA* gene cannot be considered as a factor conferring tetracycline resistance [7]. Since the first discovery of the *poxtA* gene in *Staphylococcus aureus* by a basic local alignment search tool (BLAST) search of GenBank [6], it has been also detected in enterococcal isolates from humans and animals in many countries, including China [8,9,10], Italy [11], Tunisia [12], and Pakistan [13].

*Enterococcus* is a genus of common gut commensal microorganisms present in animals and humans, and it is one of the leading causes of nosocomial infection. Moreover, enterococci in food-producing animals could transmit to humans via zoonotic transfer. Particularly, clonal complex (CC) 17 *E. faecium* is a pandemic pathogenic lineage, and its dissemination in humans, animals, and the environment interface has been documented [14]. Therefore, the emergence of CC17 *E. faecium* with resistance to multiple antimicrobial agents in the hospital environment is a great public health concern [14]. Moreover, plasmid-mediated *poxtA* gene was detected in CC17 *E. faecium* of pig origin in China, indicating an increased risk of its zoonotic transfer from animals to humans [10].

In Korea, linezolid-resistant enterococci isolated from humans and animals have been reported [2,15,16]. Linezolid resistance is mainly conferred by a 23S rRNA mutation and the *optrA* gene in clinical isolates [16], however, it is conferred by only the *optrA* gene in animal isolates [2,15]. However, the mechanisms of linezolid resistance in 8–58% of enterococci remain unknown [2,15,16]. In this study, we investigated the presence of *poxtA* gene in linezolid-resistant *E. faecium* and *E. faecalis* isolates from food-producing animals in South Korea and analyzed their molecular characteristics.

## 2. Materials and Methods

### 2.1. Bacterial Ccollection

A total of 9029 *Enterococcus* spp. (*E*. *faecium*: 3941 strains and *E. faecalis:* 5088) were obtained from 16 laboratories/centers participating in the annual Korean Veterinary Antimicrobial Resistance Monitoring System. Isolates were recovered from cattle feces (*n* = 32,516), cattle carcasses (*n* = 29,506), pig feces (*n* = 37,133), pig carcasses (*n* = 33,746), chicken feces (*n* = 21,014), chicken carcasses (*n* = 19,456), duck feces (*n* = 1470), and duck carcasses (*n* = 1733). Samples were randomly collected from farms and slaughterhouses located in all provinces of South Korea from 2008 to 2018. No more than five feces and carcasses were collected from each farm. However, the authors do not have information about the exact number of farms and slaughterhouses considered for this study.

Sample processing and enterococcal isolation were conducted as described previously [2]. Species identification was performed by matrix-assisted laser desorption ionization time-of-flight (MALDI-TOF) mass spectrometry (MS) using Vitek MS system (bioMerieux, Marcy-l’Etoile, France) or by polymerase chain reaction (PCR) assay specific for *ddl_E. faecalis_* and *ddl_E. faecium_* genes, as described previously [17]. One isolate per animal was used in this study.

### 2.2. Antimicrobial Susceptibility Testing

Antimicrobial susceptibility was evaluated by determining MICs for 16 antimicrobial agents by the broth microdilution method using commercially available Sensititre^®^ panel KRVP2F (TREK Diagnostic Systems, West Sussex, UK) according to the manufacturer’s instructions. The antimicrobials tested were ampicillin, chloramphenicol, ciprofloxacin, daptomycin, erythromycin, florfenicol, gentamicin, kanamycin, linezolid, quinupristin-dalfopristin, salinomycin, streptomycin, tetracycline, tigecycline, tylosin, and vancomycin. The reference strain *E. faecalis* ATCC 29,212 was used as the quality control strain. The MIC values were interpreted according to the Clinical and Laboratory Standards Institute (CLSI) guidelines [18].

### 2.3. Detection of Mmutations and Resistance Genes

Mutations in domain V of the 23S rRNA gene and in the genes encoding ribosomal proteins L3 (rplC) and L4 (rplD) were identified as described previously [15]. The corresponding sequences of *E. faecium* DO strain (GenBank accession number CP003583.1) and *E. faecalis* ATCC 29,212 strain (GenBank accession number CP008816.1) were used as references. The phenicol resistance gene *fexA* and oxazolidinone resistance genes *poxtA, optrA,* and *cfr* were amplified using primers as described previously [15,19]. Plasmid DNAs were extracted using the QuickGene^®^ plasmid isolation system (FUJIFILM Corporation, Tokyo, Japan).

### 2.4. Conjugation Experiment

The transferability of the plasmid carrying *poxtA* genes was assessed by the filter-mating protocol as described previously [15], using rifampicin and fusidic acid-resistant *E. faecium* BM4105RF and *E. faecalis* FA2-2 recipient strains for *E. faecium* and *E. faecalis* donor strains, respectively. Transconjugants were selected using brain heart infusion (BHI) agar (Becton Dickinson, Sparks, MD, USA) plates, supplemented with 2 µg/mL linezolid, 25 µg/mL rifampicin, and 25 µg/mL fusidic acid. All the selected transconjugants were confirmed by the detection of *poxtA* gene using PCR, and their MICs were investigated as described above.

### 2.5. Molecular Typing of poxtA-Carrying Enterococci

Pulsed-field gel electrophoresis (PFGE) was performed using the SmaI enzyme (Takara Bio Inc., Shiga, Japan), as described previously [2]. PFGE banding profiles were analyzed using Bionumerics software version 4.0 (Applied Maths, Sint-Martens-Latem, Belgium) and relatedness was calculated using the unweighted pair-group method with arithmetic averages (UPGMA) algorithm, based on the Dice similarity index. Multi-locus sequence typing (MLST) was performed as recommended on the PubMLST website (https://pubmlst.org), and allelic profiles and sequence types were determined using the *E. faecium* or *E. faecalis* MLST database (http://pubmlst.org/efaecalis/ or http://pubmlst.org/efaecium/), respectively.

## 3. Results

### 3.1. Prevalence of poxtA-Positive E. faecium and E. faecalis

Linezolid resistance (MIC = 8–16µg/mL) was found in 0.79% (94/3941) of *E. faecium* and 1.22% (62/5088) of *E. faecalis* isolates (Table 1). Amongst these, the *poxtA* gene was detected in 33.0% (31/94) of *E. faecium* and 8.1% (5/62) of *E. faecalis*. The *poxtA*-positive enterococci were mainly isolated from chicken (86.1%, 26/36), followed by duck (22%, 8/36) and cattle (5.6%, 2/36). However, no *poxtA* gene was observed in linezolid-resistant enterococci from pigs. Notably, ducks were included in the Korean Veterinary Antimicrobial Resistance Monitoring System since 2018, following our preliminary assessment in 2016. Thus, we presented the resistance profiles of duck isolates collected only in 2016 and 2018. 

### 3.2. Characterization of poxtA-Positive Enterococci 

Molecular characteristics of 31 *E. faecium* and 5 *E. faecalis* are shown in Table 2. All *poxtA*-positive enterococci were resistant to both oxazolidinone and phenicols. Furthermore, 83.9% (26/31) and 20.0% (1/5) of *E. faecium* and *E. faecalis* respectively were resistant to tetracycline. In the 31 *poxtA*-positive *E. faecium*, nine isolates co-carried both *optrA* and *fexA*, whereas four isolates co-carried either *optrA* or *fexA*. However, all *poxtA*-positive *E. faecalis* carried both *optrA* and *fexA* except for one isolate. None of the isolates carried the multi-resistance gene *cfr*. All *poxtA*-positive enterococci revealed no mutations in the genes encoding domain V of the 23S rRNA and ribosomal protein L3. However, eight *E. faecium* isolates had N130K mutation in the ribosomal protein L4.

The *poxtA* gene was transferred to recipient strains from 29.0% (9/31) and 20.0% (1/5) of *poxtA*-positive *E. faecium* and *E. faecalis* isolates, respectively (Table 2). The characteristics of transconjugants are shown in Table 3. The *poxtA* gene co-transferred with the *optrA* gene in four *E. faecium* isolates and one *E. faecalis* isolate. All the transconjugants demonstrated resistance to linezolid, chloramphenicol, and florfenicol. Additionally, we noted erythromycin resistance in each of *E. faecium* and *E. faecalis* transconjugants. Furthermore, a single *E. faecium* transconjugant exhibited resistance to tetracycline.

The *poxtA*-positive enterococci were distributed in 34 farms located in 10 provinces. A majority of the *poxtA*-positive enterococci were heterogeneous. A total of 34 pulsotypes and 25 sequence types (STs) were designed in 36 *poxtA*-positive enterococci (Table 2, Figure S1). Amongst these, six *E. faecium* strains represented five novel sequence types (STs): ST1704 (*n* = 1), ST1705 (*n* = 1), ST1706 (*n* = 1), ST1707 (*n* = 2), and ST1708 (*n* = 1). All novel STs were assigned by the PubMLST website (http://pubmlst.org/). Identical PFGE and STs (pm-11/ST237 and pm-1/ST1707) were detected in chicken from different farms (Farm V and AG, Farm G and AD). Three *E. faecium* belonging to CC17 (ST32, ST121, and ST491) were detected in two chicken feces and one cattle carcass from three different farms.

## 4. Discussion

We detected the phenicol–oxazolidinone resistance gene *poxtA* in linezolid-resistant enterococci isolated from food-producing animals. To our knowledge, we report the transferable *poxtA* gene for the first time in Korea. This study also reports the first *poxtA*-carrying isolates from duck feces and carcasses. Since the discovery of the *poxtA* gene in *Staphylococcus aureus* by a BLAST search of the GenBank [6], it has been mainly reported in enterococci from pig samples [8,9,10,11]. However, in this study, the *poxtA* gene was mainly detected in enterococci of poultry origin (chicken 86.1%, duck 22.0%). The acquisition of linezolid resistance has been known to be associated with the use of phenicols and macrolides (linked to *optrA*) or tetracyclines (linked to *poxtA*) in livestock [3,6,12]; the emergence of linezolid resistance through the acquisition of the *poxtA* gene in Korean livestock might be related to the use of these antimicrobials, which accounted for about 16–24% of all antimicrobials sales during the study period [20]. Indeed, the variation in antimicrobial use in livestock husbandry among countries could affect the selection of resistance genes.

Mutations in the domain V of the 23S rRNA gene, such as the G2576T mutation, mainly confer linezolid resistance in *Enterococcus* strains of human origin [21]; however, in this study, all *poxtA*-positive *Enterococcus* isolates lacked any mutations in the domain V of the 23S rRNA gene. These results are consistent with those of other studies on *poxtA*-carrying enterococci from environmental samples [3]. In general, although mutational changes in 23S rRNA are often associated with the use of linezolid in human infections [3,22], linezolid is not approved for use in livestock worldwide, including Korea. Thus, in the present study, the lack of 23S rRNA mutation in animal isolates might be because the use of linezolid is not prevalent. In this study, eight *E. faecium* isolates had N130K mutation in the ribosomal protein L4. We are unsure whether this mutation was involved in linezolid resistance, because we did not observe the increase of MICs for linezolid in isolates with this mutation. Thus, further studies are needed to evaluate the relationship between this mutation and linezolid resistance.

Among the 36 *poxtA*-positive enterococci, six isolates that we could not have revealed the resistance mechanisms in our previous studies [2,15] harbored *poxtA* genes. These results suggest that the *poxtA* gene could confer linezolid resistance as the sole mechanism of resistance. However, 15 *poxtA*-carrying isolates (11 *E. faecium* and 4 *E. faecalis*) co-carried the *optrA* gene with or without *fexA*. The *poxtA*- and *optrA*-co-carrying enterococci have been reported previously in China and Pakistan [8,13]. Hao et al. [8] reported that these multiple resistance gene combinations located on the same plasmid conferred higher levels of oxazolidinone resistance. Although the linezolid MICs for transconjugants with *poxtA* and *optrA* (16 µg/mL) were slightly higher than those with *poxtA* only (8 µg/mL), there was no significant difference in the linezolid MICs by the number of carried resistance genes.

The isolates of nine *E. faecium* and one *E. faecalis* transmitted their plasmids containing *poxtA* genes to *E. faecium* BM4105RF and *E. faecalis* FA2-2 recipients by conjugation, respectively. Amongst these, all isolates harboring another oxazolidinone resistance gene *optrA* co-transferred their *optrA* gene with *poxtA*. Enterococci harboring both *poxtA* and *optrA* genes from pigs, humans, and/or environmental samples have been reported in China [8], Pakistan [13], Ireland [23], and Spain [3,24]. Among these reports, the co-transfer of *poxtA* and *optrA* by conjugation was detected only in *E. faecalis* from swine in China [8] and Spain [24]. Thus, to our knowledge, this is the first report on the co-transferability of *poxtA* and *optrA* genes via conjugation in *E. faecium* isolates.

Combination analysis of molecular typing using PFGE and MLST indicated that *poxtA*-carrying isolates were heterogeneous with 34 different types. However, two types of isolates (pm-11/ST237 and pm-1/ST1707) were each detected in two different farms (Farm V and AG, Farm G and AD, respectively). Additionally, we noted isolates with the same pulsotypes and sequence types (05-CM-FM-35/05-CM-FM-36 and 06-CF-FM-42/06-BM-FM-43) or with the same sequence type and different pulsotypes (13-CF-1FC-55/13-CM-FC-56) from the same or different farms. These results suggest the emergence of clones harboring the *poxtA* gene, farm-to-farm transmission, and/or slaughterhouse contamination of *poxtA*-carrying enterococci in Korea. Notably, we had registered three novel *pstS* alleles and five ST types of *E. faecium* on the MLST database. Point mutation and recombination of housekeeping genes contribute to the clonal diversification and evolution of *E. faecium* [25]. Of note, we detected three isolates belonging to CC17 (ST32, ST121, and ST491) from two chicken fecal samples and one cattle carcass sample from three different farms. The *E. faecium* population consists of two distinct subpopulations termed clade A (the hospital-associated clade) and clade B (the community-associated clade). Clade A is responsible for the global emergence of VRE, to which CC17 *E. faecium* belongs [26]. Thus, the acquisition of linezolid resistance genes by hospital-associated clones of *E. faecium*, such as CC17, could be a great public health concern, resulting in limitations of treatment options against multidrug-resistant isolates including VRE [1,10].

In conclusion, we present the enterococci carrying the oxazolidinone and phenicol resistance gene, *poxtA*, from food-producing animals in South Korea. This study is the first report on the detection of the transferable *poxtA* gene in Korea. Moreover, three *poxtA*-positive *E. faecium* belonged to CC17, which are responsible for a significant proportion of hospital-associated infection. Our data indicate that the abuse of antimicrobials such as phenicols and tetracyclines in food-producing animals could lead to an increased risk of dissemination of the linezolid-resistant isolates in humans and animals. Therefore, the prudent use of antimicrobials and active surveillance on antimicrobial resistance are urgently needed to prevent animal-associated enterococci to become a reservoir for antimicrobial resistance.

## Figures and Tables

**Table 1:** Distribution of *poxtA*-carrying *Enterococcus faecium* and *E. faecalis* obtained from food-producing animals during 2008–2018 in South Korea.

Year	No. of Isolates (*poxtA*-Carrying Isolates/Linezolid-Resistant Isolates/Tested Isolates)									
	*E. faecium* (*n* = 3941)					*E. faecalis* (*n* = 5088)				
	Cattle	Pigs	Chickens	Ducks	Total	Cattle	Pigs	Chickens	Ducks	Total
2008	0/0/54	0/0/57	0/1/70	NT	0/1/181	0/0/32	0/11/52	0/1/17	NT	0/12/101
2009	0/0/34	0/0/108	0/0/55	NT	0/0/197	0/0/32	0/0/87	0/0/58	NT	0/0/177
2010	0/0/25	0/0/60	1/3/77	NT	1/3/162	0/0/61	0/0/88	0/0/162	NT	0/0/311
2011	0/0/72	0/0/163	0/0/87	NT	0/0/322	0/0/152	0/0/216	0/1/170	NT	0/1/538
2012	0/0/53	0/0/148	2/2/168	NT	2/2/369	0/0/80	0/1/145	0/4/213	NT	0/5/438
2013	0/0/46	0/0/127	1/3/134	NT	1/3/307	0/0/85	0/0/166	0/0/196	NT	0/0/447
2014	1/2/73	0/0/144	8/20/240	NT	9/22/457	0/0/99	0/0/164	0/2/229	NT	0/2/492
2015	1/1/51	0/0/142	7/14/166	NT	8/15/359	0/2/102	0/5/173	1/5/175	NT	1/12/450
2016	0/2/57	0/0/152	4/12/162	6/9/82	10/23/453	0/0/95	0/6/200	0/3/181	1/1/174	1/10/650
2017	0/0/83	0/0/175	0/6/131	NT	0/6/389	0/0/176	0/3/279	0/3/182	NT	0/6/637
2018	0/0/104	0/0/267	0/14/245	0/5/129	0/19/745	0/0/166	0/8/233	2/4/240	1/2/208	3/14/847
Total	2/5/652	0/0/1543	23/75/1535	6/14/211	31/94/3941	0/2/1080	0/34/1803	3/23/1823	2/3/382	5/62/5088

NT: Not tested.

**Table 2:** Characteristics of *poxtA*-carrying *Enterococcus faecium* and *E. faecalis* isolated from food-producing animals during 2008–2018 in South Korea ^a^.

Isolates	Sources of Animals	Sample	Farm ID	Slaughter-House ID	Province	Isolation Year	MICs (µg/mL)				Resistance Ggenes			Mutations			Self-Transfer	Pulso-Type	ST
							LNZ	CHL	FFC	TET	*optrA*	*fexA*	*cfr*	23s rRNA	*rplC*	*rplD*			
*E. faecium* (*n* = 31)																			
14-CF-FM-10	Chicken	Feces	A	p	Incheon	2010	16	>32	>32	128	+	+	−	WT	WT	N130K	+	pm-27	120
02-CF-FM-11	Chicken	Feces	O	G	Gyeonggi	2012	16	32	>32	>128	−	−	−	WT	WT	WT	−	pm-20	1241
03-CF-FM-12	Chicken	Feces	Z	H	Gangwon	2012	>16	>32	>32	128	+	+	−	WT	WT	N130K	−	pm-9	1166
08-CF-FM-38	Chicken	Feces	K	B	Gyeongbuk	2013	16	>32	>32	>128	+	+	−	WT	WT	WT	−	pm-6	124
02-CF-FM-15	Chicken	Feces	W	G	Gyeonggi	2014	16	>32	>32	>128	+	−	−	WT	WT	WT	+	pm-4	195
02-CF-FM-16	Chicken	Feces	Y	G	Gyeonggi	2014	16	32	>32	>128	−	−	−	WT	WT	WT	−	pm-18	124
02-CF-FM-21	Chicken	Feces	X	D	Gyeonggi	2014	16	>32	>32	>128	+	+	−	WT	WT	WT	+	pm-5	195
06-CF-FM-25	Chicken	Feces	P	F	Jeonbuk	2014	8	32	32	128	−	−	−	WT	WT	N130K	−	pm-19	1706
06-CF-FM-28	Chicken	Feces	H	F	Jeonbuk	2014	16	>32	>32	>128	+	−	−	WT	WT	WT	+	pm-3	1171
14-CF-FM-31	Chicken	Feces	I	P	Incheon	2014	16	32	>32	>128	−	−	−	WT	WT	WT	−	pm-13	32, CC17
05-BM-FM-32	Cattle	Carcasses	D	I	Chungnam	2014	8	>32	>32	≤2	−	+	−	WT	WT	WT	−	pm-14	491, CC17
05-CM-FM-35	Chicken	Carcasses	G	K	Chungnam	2014	16	32	>32	128	−	−	−	WT	WT	WT	−	pm-1	1707
05-CM-FM-36	Chicken	Carcasses	AD	K	Chungnam	2014	16	32	32	128	−	−	−	WT	WT	WT	−	pm-1	1707
04-CM-FM-39	Chicken	Carcasses	F	C	Chungbuk	2015	8	>32	>32	128	−	−	−	WT	WT	WT	−	pm-2	12
06-CF-FM-41	Chicken	Feces	S	F	Jeonbuk	2015	8	>32	>32	32	−	+	−	WT	WT	N130K	−	pm-10	195
06-CF-FM-42	Chicken	Feces	AG	F	Jeonbuk	2015	8	>32	>32	128	+	+	−	WT	WT	WT	−	pm-11	237
06-BM-FM-43	Cattle	Carcasses	V	J	Jeonbuk	2015	8	>32	>32	>128	+	+	−	WT	WT	WT	−	pm-11	237
14-CF-FM-44	Chicken	Feces	E	P	Incheon	2015	8	32	>32	>128	−	−	−	WT	WT	WT	−	pm-12	1704
04-CF-FM-51	Chicken	Feces	AE	C	Chungbuk	2015	8	32	32	≤2	−	−	−	WT	WT	WT	+	pm-7	56
06-CM-FM-52	Chicken	Carcasses	AF	F	Jeonbuk	2015	8	32	>32	>128	−	−	−	WT	WT	WT	−	pm-15	1708
14-CF-FM-53	Chicken	Feces	U	P	Incheon	2015	8	32	>32	128	−	−	−	WT	WT	WT	−	pm-26	Unidentified ^b^
04-CF-FM-55	Chicken	Feces	AA	C	Chungbuk	2016	8	32	>32	128	−	−	−	WT	WT	WT	+	pm-28	195
09-CF-FM-60	Chicken	Feces	J	A	Gyeongnam	2016	16	32	>32	>128	−	−	−	WT	WT	WT	−	pm-16	121, CC17
09-CF-FM-62	Chicken	Feces	R	A	Gyeongnam	2016	8	8	16	>128	−	−	−	WT	WT	WT	−	pm-21	240
14-CF-FM-66	Chicken	Feces	AH	P	Incheon	2016	8	>32	>32	128	−	−	−	WT	WT	N130K	−	pm-8	1705
07-DF-FM-50	Duck	Feces	T	O	Jeonnam	2016	8	32	32	2	−	−	−	WT	WT	WT	+	pm-23	120
07-DF-FM-29-1	Duck	Feces	B	N	Jeonnam	2016	8	>32	>32	128	+	+	−	WT	WT	WT	−	pm-22	157
07-DF-FM-31	Duck	Feces	B	N	Jeonnam	2016	8	8	16	>128	−	−	−	WT	WT	N130K	+	pm-17	14
07-DM-FM-20	Duck	Carcasses	M	O	Jeonnam	2016	8	32	32	128	+	+	−	WT	WT	N130K	−	pm-24	8
07-DM-FM-37	Duck	Carcasses	AC	O	Jeonnam	2016	8	32	32	2	+	+	−	WT	WT	N130K	−	pm-25	520
07-DM-FM-46-1	Duck	Carcasses	AB	N	Jeonnam	2016	8	32	>32	128	−	−	−	WT	WT	WT	+	pm-29	157
*E. faecalis* (*n* = 5)																			
02-CM-FC-24	Chicken	Carcasses	Q	D	Gyeonggi	2015	16	>32	>32	≤2	+	+	−	WT	WT	WT	−	pc-1	21
07-DM-FC-47	Duck	Carcasses	C	M	Jeonnam	2016	8	>32	>32	≤2	−	+	−	WT	WT	WT	−	pc-5	288
13-CF-FC-55	Chicken	Feces	L	E	Daegu	2018	8	>32	>32	≤2	+	+	−	WT	WT	WT	+	pc-4	834
13-CM-FC-56	Chicken	Carcasses	L	E	Daegu	2018	8	>32	>32	≤2	+	+	−	WT	WT	WT	−	pc-3	834
07-DM-FC-64	Duck	Carcasses	N	L	Jeonnam	2018	8	>32	>32	128	+	+	−	WT	WT	WT	−	pc-2	21

^a^ Abbreviations: CC, clonal complex; CHL, chloramphenicol; FFC, florfenicol; LNZ, linezolid; MICs, minimum inhibitory concentrations; ST, sequence type; TET, tetracycline; WT, wild-type; +, positive; and −, negative. ^b^ Unidentified by missing of *gdh* locus.

**Table 3:** Characteristics of *Enterococcus faecium* and *E. faecalis* transconjugants carrying *poxtA* gene ^a^.

Transconjugant	Donor Species	Donor Host	Transferred Resistance Genes			MICs (µg/mL)												
			*poxtA*	*optrA*	*fexA*	LNZ	CHL	FFC	VAN	ERY	DAP	AMP	GEN	SYN	TET	TGC	CIP	STR
CF-FM10-1BM	*E. faecium*	Chicken	+	+	+	16	>32	>32	≤2	≤1	4	2	≤128	2	≤2	≤0.12	2	≤128
CF-FM15-1BM	*E. faecium*	Chicken	+	+	−	16	>32	>32	≤2	≤1	4	2	≤128	≤2	≤2	0.25	4	≤128
CF-FM21-1BM	*E. faecium*	Chicken	+	+	+	16	>32	>32	≤2	≤1	4	2	≤128	4	≤2	≤0.12	2	≤128
CF-FM28-1BM	*E. faecium*	Chicken	+	+	−	16	>32	>32	≤2	8	4	2	≤128	4	≤2	≤0.12	2	≤128
CF-FM51-1BM	*E. faecium*	Chicken	+	−	−	8	32	>32	4	≤1	4	2	≤128	≤1	≤2	0.25	2	≤128
CF-FM55-1BM	*E. faecium*	Chicken	+	−	−	8	32	32	≤2	≤1	4	2	≤128	≤1	>128	0.25	2	≤128
DF-FM50-1BM	*E. faecium*	Duck	+	−	−	8	32	>32	4	2	4	≤1	≤128	≤1	≤2	0.25	4	≤128
DF-FM31-1BM	*E. faecium*	Duck	+	−	−	8	8	32	≤2	≤1	4	≤1	≤128	≤1	≤2	≤0.12	2	≤128
DM-FM46-1-1BM	*E. faecium*	Duck	+	−	−	8	32	>32	≤2	≤1	4	≤1	≤128	≤1	≤2	0.25	2	≤128
CF-FC55-7FA	*E. faecalis*	Chicken	+	+	+	8	>32	>32	4	8	2	≤1	≤128	8	≤2	≤0.12	1	≤128

^a^ Abbreviations: AMP, ampicillin; CHL, chloramphenicol; CIP, ciprofloxacin; DAP, daptomycin; ERY, erythromycin; FFC, florfenicol; GEN, gentamicin; LNZ, linezolid; MICs, minimum inhibitory concentrations; STR, streptomycin; SYN, quinupristin–dalfopristin; TET, tetracycline; TGC, tigecycline; VAN, vancomycin; +, positive; and −, negative.

## Supplementary Materials

The following are available online at https://www.mdpi.com/2076-2607/8/11/1839/s1, Figure S1. Dendrogram of SmaI-PFGE patterns of (a) *Enterococcus faecium* and (b) *E*. *faecalis* isolates harboring the poxtA gene from food-producing animals in South Korea. PFGE, pulsed-field gel electrophoresis pattern; ST, sequence type; +, positive; and −, negative.
