# Supplementary material for: Detection of the Phenicol–Oxazolidinone Resistance Gene poxtA in Enterococcus faecium and Enterococcus faecalis from Food-Producing Animals during 2008–2018 in Korea
Source: Microorganisms. 2020 Nov 23;8(11):1839. doi: 10.3390/microorganisms8111839 (PMC7700613; doi:10.3390/microorganisms8111839)
Supplement: Supplementary file 1 [file microorganisms-08-01839-s001.zip › microorganisms-997149-supplementary.pptx]

## Slide 1
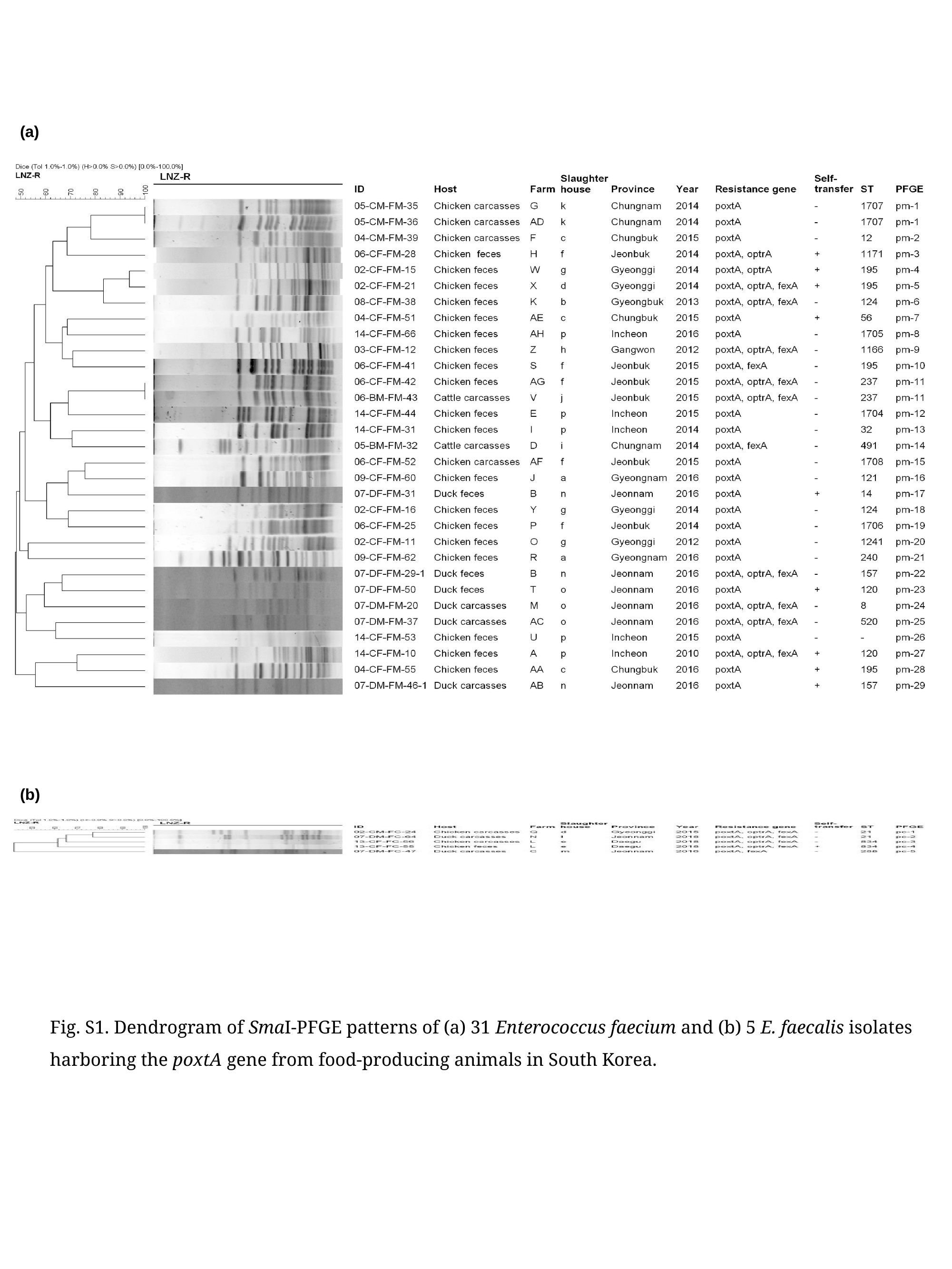

(a)
(b)
Fig. S1. Dendrogram of SmaI-PFGE patterns of (a) 31 Enterococcus faecium and (b) 5 E. faecalis isolates harboring the poxtA gene from food-producing animals in South Korea.
